# A Marker-Free *Bordetella bronchiseptica* *aroA*/*bscN* Double Deleted Mutant Confers Protection against Lethal Challenge

**DOI:** 10.3390/vaccines7040176

**Published:** 2019-11-04

**Authors:** Weicheng Ai, Zhong Peng, Fei Wang, Yue Zhang, Sisi Xie, Wan Liang, Lin Hua, Xiangru Wang, Huanchun Chen, Bin Wu

**Affiliations:** 1 State Key Laboratory of Agricultural Microbiology, College of Veterinary Medicine, Huazhong Agricultural University, Wuhan 430070, China; awc@webmail.hzau.edu.cn (W.A.); pengzhong@mail.hzau.edu.cn (Z.P.); ukf2141@webmail.hzau.edu.cn (F.W.); zhangyue0309@webmail.hzau.edu.cn (Y.Z.); xss1995@webmail.hzau.edu.cn (S.X.); liangwan521521@163.com (W.L.); hualin0@webmail.hzau.edu.cn (L.H.); wangxr228@mail.hzau.edu.cn (X.W.); chenhch@mail.hzau.edu.cn (H.C.); 2 The Cooperative Innovation Center for Sustainable Pig Production, Huazhong Agricultural University, Wuhan 430070, China; 3 Key Laboratory of Prevention and Control Agents for Animal Bacteriosis (Ministry of Agriculture), Animal Husbandry and Veterinary Institute, Hubei Academy of Agricultural Science, Wuhan 430070, China

**Keywords:** *Bordetella bronchiseptica*, *aroA*/*bscN* double deleted mutant, safety, immune efficacy, attenuated live vaccine

## Abstract

*Bordetella bronchiseptica* is a leading cause of swine respiratory disorders which depict a great threat to well-flourished porcine industry. Vaccination remains an effective way for the prevention of *B. bronchiseptica* infections, as live *B. bronchiseptica* vaccines possess many advantages compared to inactivated vaccines and/or sub-unit vaccines, however, their safety is not up to the mark. In present study, we constructed marker-free *aroA*/*bscN* double deleted *B. bronchiseptica* QH09 through two-step homologous recombination strategy. Our data showed that QH09 attenuated virulence to mice compared with the parent *aroA* deleted *B. bronchiseptica* QH0814. We also found that QH09 meets the vaccine safety standards, upon challenge in piglets, did not cause any visible clinical signs or lesions on organs. Finally, we demonstrated that vaccination of QH09 activated the systemic as well as the mucosal immunity in pigs and provided protection against lethal bacterial challenge. These findings suggest that the *aroA*/*bscN* double deleted *B. bronchiseptica* QH09 may be an effective vaccine candidate, with safety assurance of animals against *B. bronchiseptica* infections.

## 1. Introduction

*Bordetella bronchiseptica* is a small, Gram-negative, rod-shaped bacterium belonging to genus bordetella, recognized as a primary and secondary pathogenic bacterium of the upper respiratory tract in several mammals especially in dogs and in pigs [1]. In pigs, *B. bronchiseptica* mainly causes respiratory tract infections including atrophic rhinitis, pneumonia, tracheitis, and it is also a main causative agent of porcine respiratory disease complex (PRDC) [2]. These diseases are of high economic importance to the global animal industry.

Currently, vaccination is still an effective preventive remedy against *B. bronchiseptica* infections. Although inactivated vaccines have been used in pig industry for many years, their effects are limited and *B. bronchiseptica* strains could be still isolated from the nasal cavities of animals immunized with inactivated vaccines [3]. Instead, live vaccines are capable of providing more effective protection, as *B. bronchiseptica* live vaccines are capable of inducing mucosal secretory IgA (sIgA) antibodies in addition to the systemic immunity [4,5]. The live vaccines can also cause self-limiting, asymptomatic infection and therefore produce immune responses closest to those induced by natural infection [6]. However, there is a need to ensure the clinical safety of the live vaccines in piglets.

Our previous study reported a marker-free *aroA* deleted *B. bronchiseptica* vaccine which triggered a robust mucosal immune response in pigs and provides full protection against intranasal challenge [6]. In this study, we further deleted the *bscN* gene which encodes a type III secretion system (T3SS) ATPase of this *aroA* deleted strain to increase its clinical safety. The safety and protective efficiency of this marker-free *aroA/bscN* double deleted strain was assessed using piglets.

## 2. Materials and Methods

### 2.1. Bacterial Strains, Plasmids, Primers, and Culture Conditions

Bacterial strains, plasmids, and primers used in this study are listed in Table 1. *B. bronchiseptica* was grown well on Tryptic Soy Agar medium (Becton, Dickinson and Company, MD, USA) and/or in Tryptic Soy Broth (TSB) medium (Becton, Dickinson and Company, MD, USA) supplemented with 10% of new-born calf serum (NBS) at 37 °C aerobically. 1 × aromix (tryptophan, 4 µg/mL; phenylalanine, 4 µg/mL; dihydroxybenzoic acid 1 µg/mL; and para-aminobenzoic acid, 1 µg/mL) is required for the culture of *B. bronchiseptica aroA* mutant (BbΔ*aroA*). *E. coli* strains were cultured in Luria-Bertani (LB) broth (Sigma-Aldrich, MO) at 37 °C aerobically. DL-α, ε-diaminopimelic acid (DAP, 50 µg/mL) is required for the growth of *E. coli* X7213. Media contained ampicillin (AMP, 100 µg/mL) and chloramphenicol (CHL, 20 µg/mL) as required.

### 2.2. Plasmid Construction

Genomic DNA was extracted from *B. bronchiseptica* HH0809 through a TIANamp Bacteria DNA Kit (TIANGEN, Beijing, China) and was used as the template DNA for PCR assays. The upstream and downstream fragments of the *bscN* gene was amplified by PCR assays performed in a 50 µL reaction volume containing 5 µL of 10 × PCR Buffer (TAKARA, Kyoto, Japan), 4 µL of dNTP mixture (TAKARA, Japan), 4 µL of DMSO, 2 µL of template DNA, 0.5 µL of r-Taq polymerase (TAKARA, Japan), 30.5 µL of nuclease-free water (TAKARA, Kyoto, Japan), and 2 µL each of the forward primers and reverse primers listed in Table 1. The cycling conditions were 95 °C for 5 min, followed by 30 cycles of 94 °C for 1 min, 58 °C for 1 min, and 72 °C for 2 min, with a final extension 72 °C for 10 min. PCR amplification of fragments were displayed by electrophoresis on a 1% agarose gel and then purified by using a TIANgel Midi Purification Kit (TIANGEN, Beijing, China). The upstream and downstream fragments of *bscN* were cloned into pBluescript II KS/SK (+) to generate an intermediary plasmid pSKΔ*bscN*, double digested by *Kpn*I/*Sac*I. Finally, the fragment containing the upstream and downstream fragments of bscN from pSKΔ*bscN* was cloned into the suicide plasmid pRE112 (CmR) to generate the transforming plasmid pRE∆*bscN*, which was then transformed into *E. coli* X7213 for the conjugation experiment.

### 2.3. Plasmid Conjugation and Mutant Screening

Plasmid pRE∆*bscN* was conjugated from *E. coli* X7213 to the *B. bronchiseptica* QH0814, as described previously [6]. Briefly, donor strain (X7213) and receipt strain (QH0814) at mid-log phase (10% v/v) were mixed and were then inoculated on a sterilized nitrocellulose membrane plated on a TSA agar supplemented with 10% of NBS, 50 µg/mL of DAP and 1 × aromix. After incubation at 37 °C aerobically for 6–8 h, the membrane was washed using 1 mL TSB, diluted, and finally plated on TSA agar supplemented with 10% of NBS, 50 µg/mL of DAP, 1 × aromix and 20 µg/mL of chloramphenicol. The plates with putative conjugants inoculated were then incubated at 37 °C aerobically for 48 h. After conjugation, bacterial colonies with chloramphenicol resistance (CmR) were analyzed by PCR assays with primers F1/F2, B3/B6, and Cm1/Cm2 enlisted in Table 1 to confirm the occurrence of the first crossover event and the Cm cassette, respectively. Colonies with correct profile were then inoculated in TSB with 10% of NBS and 1 × aromix, shaken at 220 rpm, 37 °C aerobically for 12 h. Following that, aliquots were plated on TSA agar containing 10% of NBS and 1 × aromix and incubated at 37 °C aerobically for 24 h. All clones formed on the plates were selected to test chloramphenicol sensitivity. Chloramphenicol-sensitive colonies (CmS) were analyzed by PCR assays with primers B1/B2, B3/B6, and Cm1/Cm2 in Table 1 to confirm the occurrence of the second crossover event. The deletion of the *bscN* gene was finally confirmed by DNA sequencing. The selected mutant was passaged 30 times and the genetic stability was determined at each passage by PCR amplifying the *bscN* gene.

### 2.4. Animals and Ethic Statement

Four-week-old female BALB/c SPF mice and 25-day-old piglets were purchased from the Laboratory Animal Center of Huazhong Agricultural University (Wuhan, China) and the Pig Breeding Center of Huazhong Agricultural University (Wuhan, China), respectively. All animals were housed separately at the Laboratory Animal Center of Huazhong Agricultural University during the experiment. The use of the animals and the experiment was approved by the Research Ethics Committee at Huazhong Agricultural University (Wuhan, China) with the approval IDs HZAUMO-2018-037 for experimental infections in mice and HZAUSW-2018-012 for experimental infections in pigs. The animals were taken cared following the Guide for the Care and Use of Laboratory Animals from the Ethical Committee for Animal Experiments at Huazhong Agricultural University, Wuhan, China.

### 2.5. Virulence Studies in Mice

Four-week-old female BALB/c SPF mice (Laboratory Animal Center, Huazhong Agricultural University, Wuhan, China) were divided into 16 groups (*n* = 6 each), and were challenged intranasally (i.n.) with 50 µL of PBS containing different colony-forming units (CFU) of HH0809, QH0814, and/or QH09, or PBS alone as a control. The number of survival mice was recorded daily post challenge, and the 50% lethal dose (LD50) values for HH0809, QH0814, and QH09 were calculated by Karber’s method [10].

### 2.6. Safety Test in Piglets

Before the test, nasal, conjunctival, and rectal swabs were collected for bacteriological detection; whole blood samples were also collected for the examination of *B. bronchiseptica* antibody levels to check the *B. bronchiseptica* status. Four groups of 25-day-old piglets (*n* = 3 each, purchased from Pig Breeding Center, Huazhong Agricultural University, Wuhan, China) were challenged intratracheally with 2 mL of PBS containing 1 × 10^11^ CFU of HH0809 (Group I) or QH09 (Group III), and 3 × 10^11^ CFU of HH0809 (Group II) or QH09 (Group IV), respectively. Conditions of health and death were recorded daily for 30 days post challenge. Dead piglets were dissected in time to record lesions on the main organs. Lungs were collected for histological analysis. Scores for evaluation of clinical signs were given as described previously, i.e., normal = grade 0; slight, yet obvious = grade 1; moderate with significant symptoms = grade 2; and severe symptoms = grade 3 [6].

### 2.7. Pig Immunization and Challenge

Four groups of 25-day-old piglets (*n* = 4 each) were immunized with 2 mL of PBS containing 2.0 × 10^10^ CFU of QH09 (Group I: i.n.) or HH0809 inactivated vaccine (Group II: intramuscularly (i.m.)), and/or PBS as the control (Groups III and IV). The HH0809 inactivated vaccine was made by inactivation of 2.0 × 10^10^ CFU of HH0809 through 0.4% formalin (v/v) at 37 °C for 48–72 h. A booster immunization was given to the pigs at 14 days post primary immunization. Blood as well as the bronchoalveolar lavage fluid (BALF) collection was performed at 0, 14, and 28 days post the primary immunization to determine the level of antibodies induced by different vaccines, using ELISA method as described previously [6]. Two weeks post the booster immunization, the piglets in the immunized groups and one of the control group were challenged intratracheally with 2.0 × 10^10^ CFU of the wild type strain HH0809. Clinical signs and body temperatures were recorded daily for 30 days. Dead piglets were dissected in time for lesions record and bacteriological analysis. Survived piglets were euthanized and dissected at 30 dpc and lesions were recorded from nasal cavities and lungs.

### 2.8. ELISA Assays

Indirect ELISA assays were performed to measure the titers of anti-*Bordetella* antibodies including IgG and IgA classes, as well as IgG1 and IgG2 subclasses, as described previously [6]. Briefly, 96-well plates (Corning., Corning, USA) with adherent cell lysate of HH0809 cultured in TSB supplemented with 10% NBS were probed with serum or bronchoalveolar lavage fluid (BALF) collected from the immunized piglets. The serum and/or BALF was serially diluted in carbonate buffer (Keqian Biology, Wuhan, China) across the plate, starting at 1:40. The end-point titer was determined by comparison with the same-treated control serum or BALF. Specific classes and subclasses of antibodies were evaluated using appropriate horseradish peroxidase-conjugated goat anti-porcine antibodies (Southern Biotechnology Associates and Pharmingen, AbD Serotec, Kidlington, U K).

### 2.9. Statistics Analysis

Data was analyzed using Student’s *t*-test. ANOVA was also performed to evaluate differences between groups of animals. Statistical significance was determined as *p* values of ˂0.05 (*).

## 3. Results

### 3.1. Construction of the Marker-Free aroA/bscN Double Deletion Strain QH09

**Figure 1:**
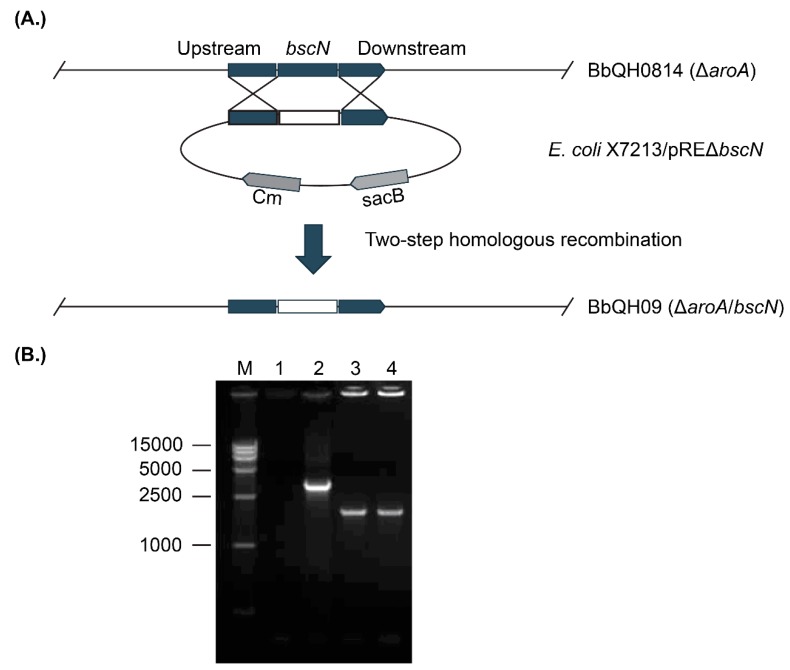
Strategy for the construction of the marker-free *aroA/bscN* double deleted *B. bronchiseptica* strain QH09. (**A**) Schematic diagram showing the strategy for the construction of the *aroA/bscN*-deletion strain. (**B**) PCR determination of the *bscN*-deletion in the *aroA*-deletion strain QH0814. M: DL 15,000 DNA marker; Lane 1: ddH_2_O as the negative control; Lane 2: QH0814 as the positive control (2661-bp); Lanes 3–4: *bscN*-deletion (2120-bp).

To generate the marker-free *aroA/bscN* double deleted strain QH09, conjugation assay was performed between *E. coli* X7213 which contains the suicide plasmid pRE-Δ*bscN* and the *aroA*-deleted *B. bronchiseptica* QH0814. Ten single-crossover transconjugants capable of growing on CHL-supplemented agars were selected for determination of PCR assays with primers F1/F2, B3/B6, and Cm1/Cm2 after conjugation. Since the presence of the integrated plasmid, PCR assays using the genomic DNA of the putative *B. bronchiseptica* single-crossover transconjugants as a template yielded a 2661-bp product for the wild-type *bscN*, a 2120-bp product for the *bscN* mutant, and a 920-bp product for the Cm box. After the second crossover, PCR assays using the genomic DNA of the putative *B. bronchiseptica* mutants as a template only yielded 2120-bp product for the *bscN* mutant; the 2661-bp product (*bscN* gene) and the 920-bp product (Cm gene) were missing (Figure 1). DNA sequencing further confirmed that the deleted fragment was *bscN*, stability of the deletion evident from repeat PCR assays (Figure S1 in Supplementary Materials).

### 3.2. QH09 Showed Attenuated Virulence and Capacity of Colonization in Mice

To test the virulence attenuation of QH09, the LD_50_ value of the strain was calculated and compared with that of the parent strain QH0814 and the wild type strain HH0809. The results revealed that the LD50 of QH09 was 4.17 × 10^8^ CFU, which attenuates approximately 20-fold compared to that of QH0804 (1.90 × 10^7^ CFU), and approximately 225-fold to that of HH0809 (1.85 × 10^6^ CFU).

### 3.3. QH09 Was Safe to Piglets

**Figure 2:**
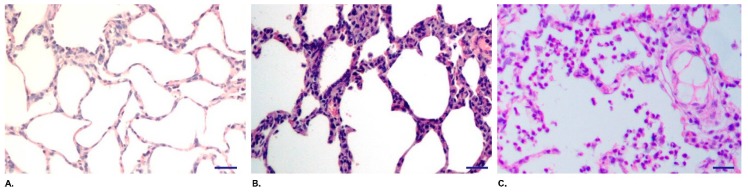
Histological analysis on pig lungs challenged with QH09, HH0809, and PBS in the pig safety tests (bar 100 μm). Panel A is a lung histological section from a pig challenged with PBS; Panel B is a lung histological section from a pig challenged with QH09 at 3 × 10^11^ CFU; Panel C is a lung histological section from a pig challenged with HH0809 at 1 × 10^11^ CFU.

Animal experiments showed that piglets challenged with either 3 × 10^11^ CFU or 1 × 10^11^ CFU of QH09 showed slight cough, deep breath, little depression, decreased appetite, and increased temperature (4041 °C), but all signs disappeared by 24–48 h (Table 2). No death was observed during the 30 days post infection. Dissection of the challenged piglets at 30 dpc did not reveal any observable lesions in lungs, tracheae, nasal cavities and other respiratory organs. However, the piglets challenged with the wild type strain HH0809 at 3 × 10^11^ CFU exhibited severe clinical symptoms. The piglets became pyrexic (41–42 °C) within 24 dpc and the high body temperature lasted during the whole observation period. The other signs included labored breathing, cough, serious depression, appetite losing, and exacerbated signs including open mouth-breathing, red and swollen nasal mucosa, and disordered coats were also observed from 2 dpc. Mortality occurred from 3 dpc and all piglets died at 7 dpc. Before death, the piglets showed decreased temperature and visible symptoms of skin cyanosis at abdomen. Piglets challenged with HH0809 at 1 × 10^11^ CFU displayed similar signs; two piglets died at 6 dpc and 7 dpc; the remaining one became thin and stiff. The gross lesions in the piglets died from an infection of HH0809 included hydropericardium and pleural effusion, edematous lungs with areas of hemorrhage and necrosis on their surfaces, edematous kidneys and spleens with hemorrhagic points on the surfaces, and swollen lymph nodes; there were bleeding spots in pericardium and epicardium, accompanied by fibrous pericarditis; the lobes of the lungs have sticky appearance towards intrathoracic wall; the tracheas were full of foamy mucus, the mucosa were edematous, congestive, and hemorrhagic. Histological check on lungs revealed that the alveolar walls of the pigs challenged with HH0809 broken; the alveolar epithelial cells were proliferous; there were many inflammatory cellular infiltrations in the lung tissue and there was serous exudation in alveolar cavities (Figure 2C). In comparison, damages on lung tissues of pigs challenged by QH09 are much slighter. The alveolar walls were intact; there was no exudation in alveolar cavities (Figure 2B). There were no visible lesions in the brains and other organs.

### 3.4. QH09 Induced High Levels of Antibodies and Conferred Protection Against Lethal Challenge

**Figure 3:**
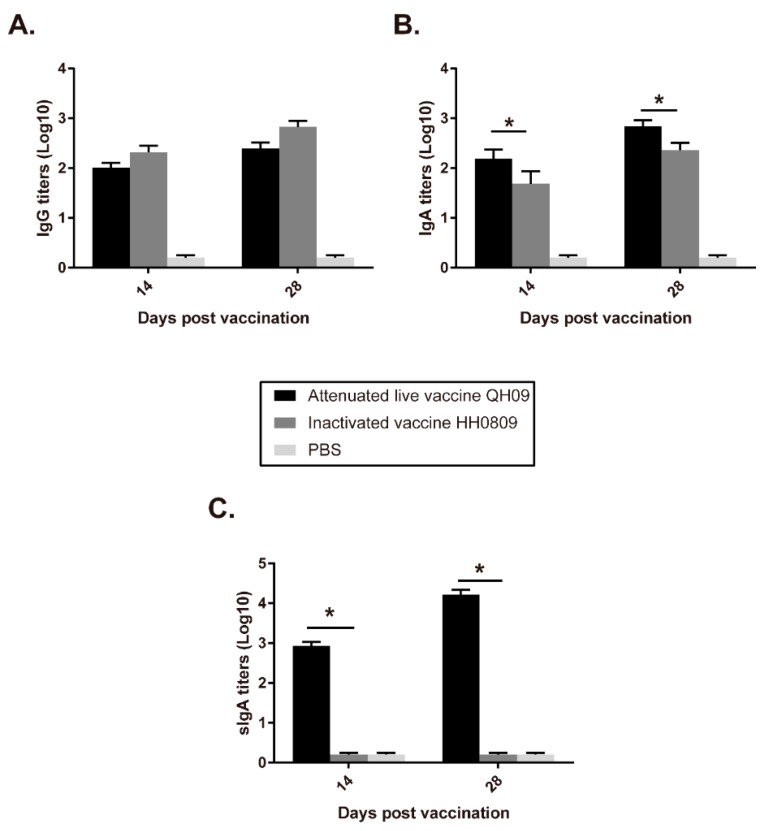
ELISA titers of IgG in serum (**A**), IgA in serum (**B**), sIgA in BALF (**C**), in piglets induced by the attenuated live vaccine (QH09), the inactivated vaccine (HH0809), and PBS. “*” *p* < 0.5.

Results of ELISA assays showed that the titers of IgG, IgA, sIgA, IgG1, and IgG2a in piglets receiving live (QH09) or inactivated (HH0809) vaccines were significantly increased after immunization (Figure 3 and Table 3). The serum IgG titer induced by the attenuated vaccine QH09 was lower than that induced by the inactivated vaccine HH0809 (Figure 3A). However, the IgA titer in the BALF and serum induced by QH09 was significantly higher than that induced by HH0809 (Figure 3B). In particularly, a high level of sIgA was detected in the BALF from pigs immunized with QH09, but there was almost no sIgA titer detected in the BALF from the HH0809-immunized group (Figure 3C).

**Figure 4:**
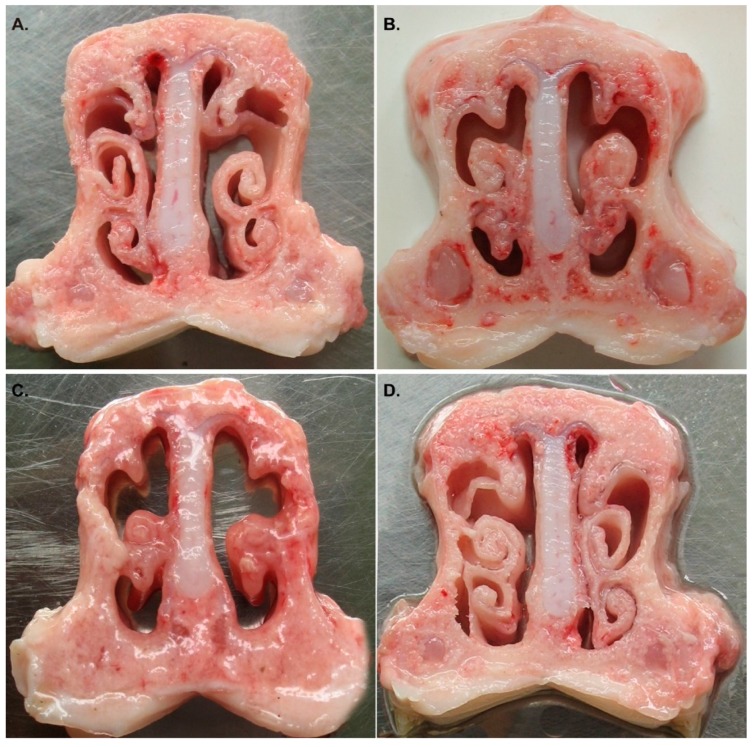
Lesions on the nasal cavities of pigs immunized with different vaccines after infection. (**A**) lesions on the nasal cavities of pigs immunized with the attenuated live vaccine (QH09); (**B**) lesions on the nasal cavities of pigs immunized with the inactivated vaccine (HH0809); (**C**) lesions on the nasal cavities of pigs immunized with PBS; (**D**) the nasal cavities of the healthy pigs.

The protective ability of QH09 was tested and compared to that of the HH0809 inactivated vaccine by infection of HH0809 (2.0 × 10^10^ CFU) during the fifth week post-first immunization. After challenge, the piglets received PBS showed serious illness, including long last (≥7 days post infection), high temperature (40.5–41 °C), disordered coats, labor breathing, serious depression, and appetite losing. Two of the piglets died at 5 dpc and 7 dpc, the remaining two showed slow growth and finally became stiff. After dissection, the tracheae of the dead piglets were full of foamy mucus and the mucosal surface was edematous, congestive, and hemorrhagic; the lungs also showed same appearance with caseous exudate. Dissection on the survived piglets on the 30 dpc showed that nasal turbinates were withered and disappeared (Figure 4). The piglets immunized with the HH0809 inactivated vaccine also showed increased temperature (>40 °C), but the symptoms only lasted 5 dpc and then recovered. Mild clinical signs were observed as compared to the piglets immunized with PBS, one died piglet after infection exhibited edematous, congestive, and hemorrhagic lung, with some caseous exudate on it. Dissection of the survival piglets showed that the turbinates of two piglets also showed visible deformation (Figure 4). Piglets given a vaccination of QH09 showed higher temperature (approximately 40.5 °C) within the 5 days post challenge, but they recovered at day 6 post challenge. Only one piglet showed the difficulty in breathing, the other three showed slight coughs, but all of them recovered later. No death was observed and no appearance of visible lesions on the lungs and nasal cavities after dissection (Figure 4).

## 4. Discussion

Most pathogens enter the body through mucosal surfaces and, therefore, mucosal vaccines (mostly are live attenuated vaccines) that target the respiratory, gastrointestinal, or urogenital tracts are attractive as they stimulate local protection against infections [7]. This is really true for the vaccine against the infection caused by the respiratory pathogenic bacterium *B. bronchiseptica* which can efficiently colonize respiratory mucosa, as an attenuated live *B. bronchiseptica* vaccine often induces immunity and provides effective and long-lasting protection superior to those achieved by an inactivated vaccine and/or a sub-unit vaccine [3,6,11,12,13]. In addition, another advantage of attenuated live *B. bronchiseptica* vaccine is that could be administrated directly through nasal cavity, making vaccination more convenient. However, the greatest concern for an attenuated live vaccine is the safety of the animals after administration. In one of our previous studies, we developed an attenuated live *B. bronchiseptica* vaccine by knocking out its *aroA* gene, which encodes the 5-enolpyruvyl-shikimate-3-phosphate synthase that is crucial for the bacterial in vivo survival and growth [6]. While such strategy enables the attenuation without compromising the antigenicity, this *B. bronchiseptica* mutant might be not safe enough to keep all virulence factor-encoding genes inactive. Therefore, we knocked-out the *bscN* gene of the *aroA*-deletion vaccine we developed in our previous study [6], to increase the safety of the vaccine.

The *bscN* gene encodes an ATPase that provides energy for the secretion of effector proteins and BscN protein is required for the function of the T3SS apparatus [14]. It has been found that the in-frame deletion of *bscN* leads to decreased cytotoxicity of *B. bronchiseptica* to mammalian cells in vitro and the *bscN* deletion strain is defective for long-term tracheal colonization in rats [14]. These findings suggest that *bscN* involves in the virulence of *B. bronchiseptica*. In this study, the LD50 of the *aroA*/*bscN* double deletion *B. bronchiseptica* strain QH09 attenuates approximately 20-fold compared to that of QH0804 (4.17 × 10^8^ CFU vs. 1.90 × 10^7^ CFU). The clinical signs in piglets induced by the *bscN* deletion strain (Table 2) are less severe than those of parent strain, reported in our previous study [6]. All these findings suggest that the *aroA*/*bscN* double deletion strain is safer than the *aroA* deletion strain as a live vaccine candidate.

In addition to safety, the other parameters to determine an ideal vaccine candidate are the effectiveness and convenient administration [13]. While several types of anti-*B. bronchiseptica* vaccines such as the inactivated vaccines and/or the subunit vaccines including the recombinant OMPs or the novel adjuvant BfcA are commercially available or reported in laboratory phase [10,15,16], attenuated live vaccines are still proposed to be the preferable vaccines [17]. First, compared to the inactivated *B. bronchiseptica* vaccines and/or subunit vaccines which are often administered intramuscularly, the attenuated live *B. bronchiseptica* vaccines are administered through intranasal inoculation, which represents an easier way for administration [6,17,18,19]. Second but the most important reason, administration of the attenuated live *B. bronchiseptica* vaccine is capable of inducing mucosal immune response [6]. It has been documented that mucosal immune responses are most efficiently induced by administration of vaccines onto mucosal surfaces, whereas injected vaccines are generally poor inducers of mucosal immunity [4]. The production of sIgA represents a hallmark of the mucosal immune response [5,20]. In our study, immunization of the attenuated live *B. bronchiseptica* vaccine QH09 induced the production of IgG, and IgA antibodies in immunized pigs (Figure 4), suggesting that QH09 can induce immune responses effectively. In particular, administration of the attenuated live *B. bronchiseptica* vaccine QH09 induced a production of sIgA which was not detected in the BALF of the inactivated vaccine immunized-pigs (Figure 3), suggesting that vaccination with QH09 can also induce the mucosal immunity. It has been described that the sIgA antibodies play an important role in limiting the overgrowth of commensal microbes, and they also protect the host by binding to the surface of luminal microbes and toxins to prevent them from attaching to epithelial cells [5]. Therefore, the induction of the mucosal immunity and the production of the sIgA antibodies are crucial in order to prevent subsequent bacterial infections [11,17]. It is worthy of note that all of the results obtained from the present study are in agreement with those from the pigs immunized with the *aroA* deleted *B. bronchiseptica* QH0814 [6]. These findings suggest that vaccination of QH09 induces immune responses effectively and a deletion of the *bscN* gene does not alter its immune-stimulant efficacy. This could be also supported by the results from the protective test, as the vaccination of QH09 could protect the pigs against the lethal challenge of the wild type strain HH0809 and decrease the lesions on the turbinates (Figure 4).

## 5. Conclusions

To be concluded, an *aroA*/*bscN* double deleted *B. bronchiseptica* was generated in this study; this mutant showed lower capacity of colonization and virulence than the parent *aroA* deleted strain which encompasses all safety characteristics without compromising its efficacy. Vaccination of this *aroA*/*bscN* double deleted *B. bronchiseptica* activated the systemic and mucosal immunity in pigs and provided protection against lethal challenge, which represents an effective vaccine against *B. bronchiseptica* infections. 

## Figures and Tables

**Table 1:** Bacterial strains, plasmids, and primers used in this study.

Bacterial Strains	Characteristics	Source
*B. bronchiseptica* HH0809	Wild type isolate from a pig with atrophic rhinitis	[7,8]
*B. bronchiseptica* QH0814	Unmarked *aroA* deletion of HH0809	[6]
*B. bronchiseptica* QH09	Unmarked *bscN* deletion of QH0814	This study
*E. coli* X7213	Thi-1 thr-1 leuB6 fhuA21 lacY1 glnV44 △asdA4 recA1 RP4 2-Tc::Mu[λpir] Kmr	[9]
**Plasmids**		
pBluescript II KS/SK (+)	oriColE1 oriF1(+) bla lacZa	Our collection
pRE112 (CmR)	oriT oriV sacB1, CmR, counterselectable suicide plasmid	[9]
pSKΔ*bscN*	*bscN* upstream and downstream fragment amplified from QH0814 ligated into pBluescript II KS/SK (+)	This study
pREΔ*bscN*	*bscN* upstream and downstream fragment from pSKΔ*bscN* ligated into pRE112	This study
**Primers**		
F1	5′- CCCCCGCACATTTCCGAACTTC -3′	Part of flagellin (237 bp)
F2	5′- AGGCTCCCAAGAGAGAAAGGCTT -3′	
B1	5′- CGGGAATTCATGCGTCAGTACCACTACATCAC -3′ (*EcoR* I)	Part of *bscN* (1334 bp)
B2	5′- GCTAAGCTTTAGGATTCGGGTCCGATGATTTCAG -3′ (*Hind* III)	
B3	5′- TAA GGTACC GCGCTTGCGCTGGTGCTGTC -3′ (*Kpn* I)	*bscN* upstream flank (1148 bp)
B4	5′- TCA GAATTC ACGCGCACCCCCAGCTCC -3′ (*EcoR* I)	
B5	5′- TGTGAATTC GACGGCCACATCGTGCTCT -3′ (*EcoR* I)	*bscN* downstream flank (980 bp)
B6	5′- GGAGAGCTCCCTTCGCTTTCTCCTGTTCCAT -3′ (*Sac* I)	
Cm1	5′- TAAATACCTGTGACGGAAGAT -3′	Cm resistant gene (700 bp)
Cm2	5′- TATCACTTATTCAGGCGTAGC -3′	

**Table 2:** Scores indicating clinic signs in pig safety test.

Group	Piglets ID	24 hpc		48 hpc		First Week	
		Appetite ^a^	Mood ^a^	Cough ^a^	Appetite ^a^	Spirit ^a^	Cough ^a^
QH09 (3 × 10^11^ CFU)	1	1	1	0	0	0	0
	2	1	1	0	0	0	0
	3	1	1	0	0	0	0
QH09 (1 × 10^11^ CFU)	1	1	1	0	0	0	0
	2	1	1	0	0	0	0
	3	1	1	0	0	0	0
HH0809 (3 × 10^11^ CFU)	1	3	3	3	3	3	3
	2	3	3	3	3	3	3
	3	3	3	3	3	3	3
HH0809 (1 × 10^11^ CFU)	1	3	3	3	3	3	3
	2	3	3	3	3	2	2
	3	3	3	3	3	2	2
PBS	1	0	0	0	0	0	0
	2	0	0	0	0	0	0
	3	0	0	0	0	0	0
Mean	QH09 (3 × 10^11^ CFU)	1.0 ± 0.00	1.0 ± 0.00	0	0	0	0
	QH09 (1 × 10^11^ CFU)	1.0 ± 0.00	1.0 ± 0.00	0	0	0	0
	HH0809 (3 × 10^11^ CFU)	3.0 ± 0.00	3.0 ± 0.00	3.0 ± 0.00	3.0 ± 0.00	3.0 ± 0.00	3.0 ± 0.00
	HH0809 (1 × 10^11^ CFU)	3.0 ± 0.00	3.0 ± 0.00	3.0 ± 0.00	3.0 ± 0.00	2.3 ± 0.47	2.3 ± 0.47
	PBS	0	0	0	0	0	0

^a^ Clinical signs were scored as: normal (grade 0); slight, yet obvious (grade 1); moderate with significant symptoms (grade 2); and severe symptoms (grade 3).

**Table 3:** IgG1 and IgG2a tiers induced by different vaccines at different days post vaccination.

Antibodies (Log10)	Attenuated Live Vaccine (QH09)		Inactivated Vaccine (HH0809)	
	14 Days (OD630)	28 Days (OD630)	14 Days (OD630)	28 Days (OD630)
IgG1	1.83 ± 0.17	2.20 ± 0.21	3.83 ± 0. 0.30	4.47 ± 0.17
IgG2a	2.60 ± 0.37	2.97 ± 0.17	3.13 ± 0.11	3.54 ± 0.15
IgG1/IgG2a	0.70	0.74	1.22	1.26

Baseline titers are zero.

## Supplementary Materials

The following are available online at https://www.mdpi.com/2076-393X/7/4/176/s1. Figure S1: PCR assays indicated the genetic stability of the *aroA*/*bscN* double deleted *B. bronchiseptica* QH09. The bacterium was passaged on blood-TSA for 50 passages and strains were selected every five passages to determine the deletion of the *bscN* gene PCR assays with primers B3/B6 listed in Table 1. M1: DL 15,000 DNA marker; M2: DL 2000 DNA marker; Lane 1: ddH_2_O as a negative control; Lane 2: the wild type strain HH0809 as a positive control; Lanes 3–12: colonies of OH09 selected from the 5th, 10th, 15th, 20th, 25th, 30th, 35th, 40th, 45th, and 50th passages, respectively.
