# Supplementary figures and images for: A Marker-Free Bordetella bronchiseptica aroA/bscN Double Deleted Mutant Confers Protection against Lethal Challenge
Source: Vaccines (Basel). 2019 Nov 4;7(4):176. doi: 10.3390/vaccines7040176 (PMC6963861; doi:10.3390/vaccines7040176)

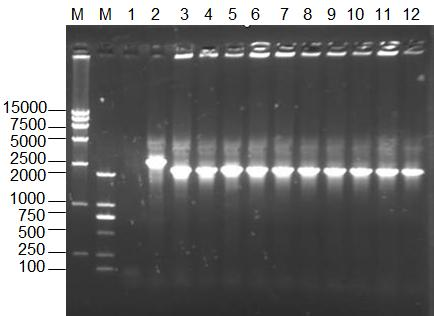

Supplement: Supplementary file 1 [file vaccines-07-00176-s001.zip › vaccines-555233-supplementary.tif]
